# The full repertoire of *Drosophila* gustatory receptors for detecting an aversive compound

**DOI:** 10.1038/ncomms9867

**Published:** 2015-11-16

**Authors:** Jaewon Shim, Youngseok Lee, Yong Taek Jeong, Yonjung Kim, Min Goo Lee, Craig Montell, Seok Jun Moon

**Affiliations:** 1 Department of Oral Biology, BK21 PLUS Project Yonsei University College of Dentistry, Yonsei-ro 50-1, Seodaemun-gu, Seoul 120-752, Korea; 2 Division of Functional Food Research, Korea Food Research Institute, Seongnam, Gyeonggi-do 463-746, Korea; 3 Department of Bio and Fermentation Convergence Technology, Kookmin University, Seoul 136-702, Korea; 4 Department of Pharmacology, Brain Korea 21 PLUS project for Medical Sciences Yonsei University College of Medicine, Yonsei-ro 50-1, Seodaemun-gu, Seoul 120-752, Korea; 5 Department of Molecular, Cellular, and Developmental Biology, Neuroscience Research Institute, University of California, Santa Barbara, California 93106, USA

## Abstract

The ability to detect toxic compounds in foods is essential for animal survival. However, the minimal subunit composition of gustatory receptors required for sensing aversive chemicals in *Drosophila* is unknown. Here we report that three gustatory receptors, GR8a, GR66a and GR98b function together in the detection of L-canavanine, a plant-derived insecticide. Ectopic co-expression of *Gr8a* and *Gr98b* in *Gr66a*-expressing, bitter-sensing gustatory receptor neurons (GRNs) confers responsiveness to L-canavanine. Furthermore, misexpression of all three *Gr*s enables salt- or sweet-sensing GRNs to respond to L-canavanine. Introduction of these *Gr*s in sweet-sensing GRNs switches L-canavanine from an aversive to an attractive compound. Co-expression of GR8a, GR66a and GR98b in *Drosophila* S2 cells induces an L-canavanine-activated nonselective cation conductance. We conclude that three GRs collaborate to produce a functional L-canavanine receptor. Thus, our results clarify the full set of GRs underlying the detection of a toxic tastant that drives avoidance behaviour in an insect.

## Abstract (web summary)

It remains unclear whether any set of the 68 gustatory receptors expressed in *Drosophila* comprise a cation channel that responds to an aversive chemical. Here the authors identify three gustatory receptors that are both necessary and sufficient to form a channel that confers sensitivity to a noxious tastant.

## Graphical abstract

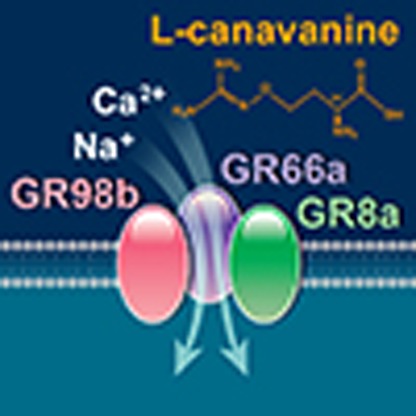

Taste is critical for evaluating food quality. Dedicated sensory cells are devoted to each taste modality, sending sensory information to higher brain centres that direct either attraction or aversion^1^. Because many bitter and other avoidance compounds are toxic, rapid and accurate detection of these compounds is an important defense in many herbivorous animals.

In *Drosophila*, several types of membrane proteins participate in the perception of aversive chemicals. At least two transient receptor potential (TRP) channels, TRPA1 and TRPL, function in the sensation of deterrent compounds^2^,^3^,^4^. However, the detection of most aversive tastants are thought to be accomplished through members of the gustatory receptor (GR) family, which encodes 68 proteins^5^,^6^,^7^,^8^. Fly GRs are unrelated to mammalian taste receptors, which are G-protein coupled receptors, but they are distantly related to the *Drosophila* olfactory receptors (ORs)^7^,^9^,^10^,^11^,^12^,^13^,^14^. Heteromeric OR complexes comprise odorant-gated-cation channels^15^,^16^,^17^, and it is reported that insect fructose receptors consist of single GR subunits, which form nonselective cation channels^18^.

The *Drosophila* GRs that respond to noxious compounds consist of multiple subunits. Based on *in vivo* loss-of-function studies, three GRs are broadly tuned (GR32a, GR33a and GR66a) and function in the detection of a wide range of avoidance compounds^19^,^20^,^21^. In addition, other GRs, such as GR8a, GR47a and GR93a, are narrowly tuned and required for sensing L-canavanine, strychnine and caffeine, respectively^22^,^23^,^24^.

L-canavanine is a plant-derived analogue of the amino acid L-arginine^25^,^26^,^27^, and ingestion of this compound is lethal to fruit flies and many other insects because it incorporates into proteins in place of L-arginine. We previously reported that GR8a and GR66a are required for L-canavanine detection^22^. However, co-expression of *Gr8a* and *Gr66a* in sweet-sensing GRNs does not confer responsiveness to L-canavanine. Currently, the minimum subunit composition of the L-canavanine receptor or any of the other GR complexes that respond to aversive compounds are unknown. It is also unclear if these heteromultimeric GRs are cation channels.

In this study, we perform an RNA interference (RNAi) screen to identify the complete set of receptors involved in L-canavanine detection. As expected, knockdown of *Gr8a* and *Gr66a* impairs L-canavanine avoidance behaviour. In addition, we find that suppressing expression of one additional gene (*Gr98b*) disrupts L-canavanine avoidance. We deleted *Gr98b*, which eliminates the behavioural repulsion and action potentials in response to L-canavanine. Introduction of *Gr8a*, *Gr66a* and *Gr98b* together in sweet-sensing GRNs or low salt-sensing GRNs, endows these cells with the ability to respond to L-canavanine. Moreover, ectopic expression of these GRs in sweet-sensing GRNs switches the flies' innate L-canavanine aversion to attraction. Ectopic expression of *Gr8a*, *Gr66a* and *Gr98b* in S2 tissue culture cells confers L-canavanine-dependent currents. Our findings define the first heteromultimeric GR complex that is required and sufficient for conferring sensitivity to an aversive compound.

## Results

### Screening for receptors required for L-canavanine detection

Our previous findings show that GR8a and GR66a are required but not sufficient for L-canavanine detection^22^. Therefore, we performed an RNAi screen to address a potential requirement for other GRs, by interrogating the full set of 58 available *UAS*–*Gr* RNAi lines. We also knocked down 13 genes encoding ionotropic receptors (IRs) that were expressed in GRNs^28^,^29^. We crossed these RNAi lines to flies that expressed *Dicer* (*UAS*–*Dcr2*) and the *Gr33a*–*GAL4* driver, which is expressed in GRNs that respond to aversive compounds^21^. All of the progeny were viable and appeared healthy.

**Figure 1:**
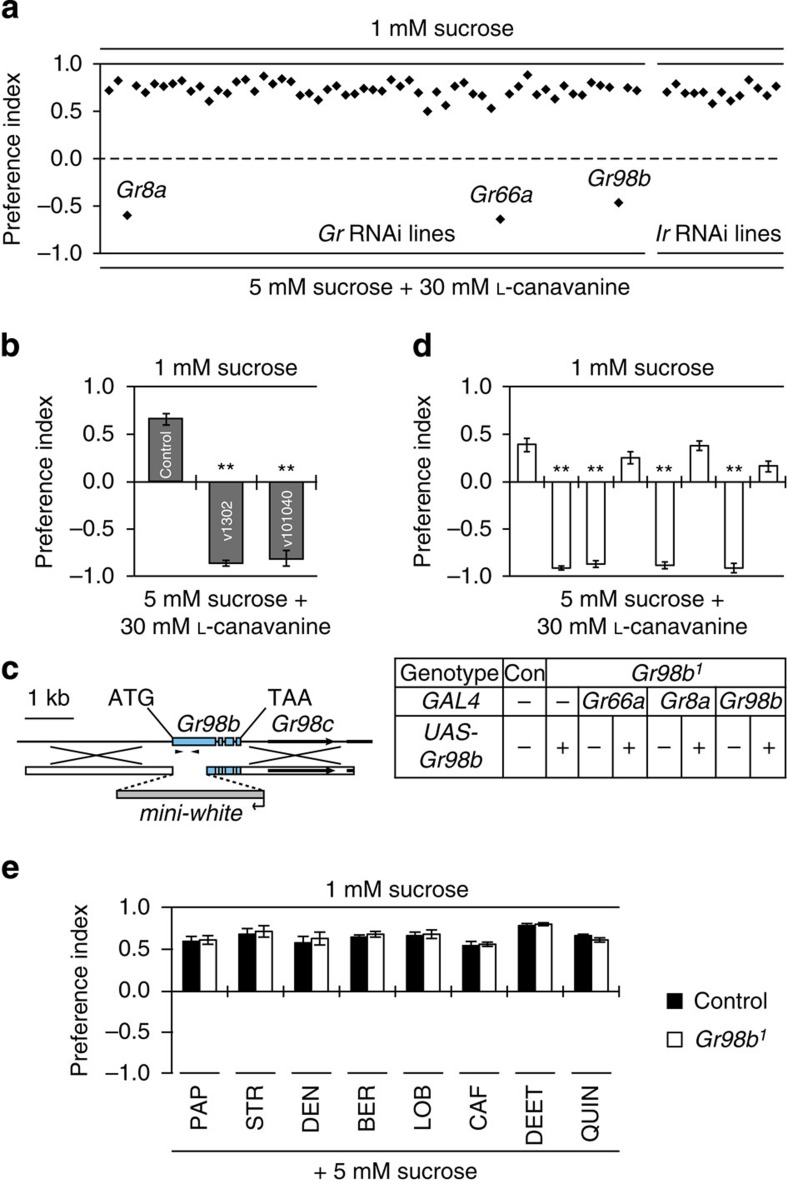
Identification of taste receptors required for L-canavanine avoidance. (**a**) An RNAi screen of 58 *UAS–Gr* RNAi lines and 13 *UAS*–*Ir* RNAi lines for defects in L-canavanine avoidance. We drove expression of the RNAi lines using the *Gr33a*–*GAL4*, and included *UAS*–*Dcr2* (*Dicer2*) to improve the efficacy of the RNAi. The dashed line indicates no preference. See the Methods section (fly stocks) for the list of stocks screened. (**b**) Two-way choice assays after knocking down *Gr98b* using two different RNAi lines. The control consisted of *UAS*–*Dcr2*;*Gr33a*–*GAL4* flies without the RNAi transgenes. RNAi stock numbers (VDRC) are indicated within the bars. *n*=5 for each genotype. **P<0.01 (analysis of variance (ANOVA) with *post hoc* Tukey test). (**c**) Cartoon showing the strategy for creating the *Gr98a*^*1*^ allele by ends-out homologous recombination. The arrowheads indicate the genomic PCR primers used to confirm the *Gr98b* deletion. A 543 bp band present in control flies was absent in *Gr98b*^*1*^. (**d**) Two-way choice assays to test whether *Gr98b*^*1*^ displayed a deficit in L-canavanine avoidance. To test for rescue of the *Gr98b*^*1*^ phenotype, we expressed the *Gr98b* cDNA in the *Gr98b*^*1*^ background using the *Gr66a*–*GAL4*, the *Gr8a*–*GAL4* and the *Gr98b*–*GAL4*. *n*=5 for each genotype. **P<0.01 (ANOVA with *post hoc* Tukey test). (**e**) Two-way choice assays to test for avoidance of *Gr98b*^*1*^ flies in response to the indicated bitter chemicals. The flies were given a choice between 1 mM sucrose and 5 mM sucrose plus the following aversive compounds: 0.5 mM papaverine (PAP), 0.5 mM strychnine (STR), 0.1 mM denatonium (DEN), 0.05 mM berberine (BER), 0.1 mM lobeline (LOB), 5 mM caffeine (CAF), 0.2% N,N-diethyl-m-toluamide (DEET), and 0.5 mM quinine (QUIN). *n*=4–7 for each genotype. All data are mean±s.e.m.

To assess L-canavanine avoidance, we performed two-way choice behavioural assays. Given a choice between 1 mM sucrose and 5 mM sucrose mixed with 30 mM L-canavanine, wild-type flies strongly avoid the higher sugar laced with L-canavanine^22^. As expected, knockdown of either *Gr8a* or *Gr66a* dramatically reduced L-canavanine avoidance^22^ (Fig. 1a). In addition, we found that RNAi-mediated suppression of one other receptor (*Gr98b*) also reduced L-canavanine avoidance dramatically (Fig. 1a; v101040 line). Introduction of all other *UAS*–*Gr* or *UAS–Ir* RNAi lines had no impact on L-canavanine avoidance (Fig. 1a). We tested an additional *UAS–Gr98b* RNAi line (v1302), which produced the same phenotype as the first line (v101040; Fig. 1b). Thus, GR98b was an additional candidate receptor critical for detecting L-canavanine.

### Mutation of *Gr98b* impaired L-canavanine detection

**Figure 2:**
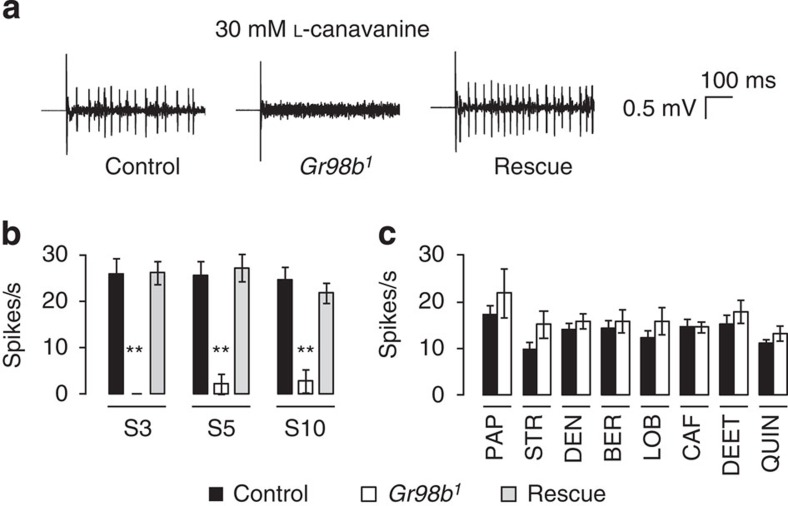
Dependence of L-canavanine induced action potentials on *Gr98b*. (**a**) Action potentials elicited in S6 sensilla of control (*w*^*1118*^), *Gr98b*^*1*^ and rescue flies (*Gr66a*–*GAL4*/*UAS*–*Gr98b*;*Gr98b*^*1*^) in response to 30 mM L-canavanine. (**b**) Mean frequencies of action potentials upon exposure to 30 mM L-canavanine. Indicated are the genotypes and sensilla tested (S3, S5 and S10), *n*=10–12 for each genotype. (**c**) Mean frequencies of action potential induced in S6 sensilla in response to the indicated bitter chemicals (1 mM PAP, 1 mM STR, 1 mM DEN, 0.1 mM BER, 1 mM LOB, 10 mM CAF, 0.2% DEET and 1 mM QUIN). *n*=10–21 for each genotype. All data are mean±s.e.m. **P<0.01 (analysis of variance with *post hoc* Tukey test).

To confirm a role for *Gr98b* for L-canavanine repulsion, we generated a mutation by ends-out homologous recombination (Fig. 1c). The *Gr98b*^*1*^ mutation deleted the region encoding the N-terminal 233 out of 403 residues. The mutant flies were homozygous viable and fertile. Consistent with the RNAi experiments, the *Gr98b*^*1*^ flies failed to avoid L-canavanine (Fig. 1d). We fully rescued L-canavanine avoidance in the *Gr98b*^*1*^ flies by expressing a wild-type *Gr98b* transgene (*UAS*–*Gr98b*) under the control of the *Gr66a* (*Gr66a*–*GAL4*), the *Gr8a* (*Gr8a*–*GAL4*) or the *Gr98b* promoter (*Gr98b*–*GAL4*; Fig. 1d). L-canavanine-induced action potentials were also abolished in *Gr98b*^*1*^ flies, and the defect was rescued by expressing the wild-type *Gr98b* transgene (*UAS*–*Gr98b*) using the *Gr66a*–*GAL4* (Fig. 2a,b). In contrast to the effects on sensing L-canavanine, *Gr98b*^*1*^ flies displayed robust aversion and electrophysiological responses to papaverine, strychnine, denatonium, berberine, lobeline, caffeine, N,N-diethyl-m-toluamide (DEET) and quinine (Figs 1e and 2c). These data indicated that GR98b was required for the detection of L-canavanine, and was narrowly tuned.

### Endowing L-canavanine responsiveness to bitter-sensing GRNs

**Figure 3:**
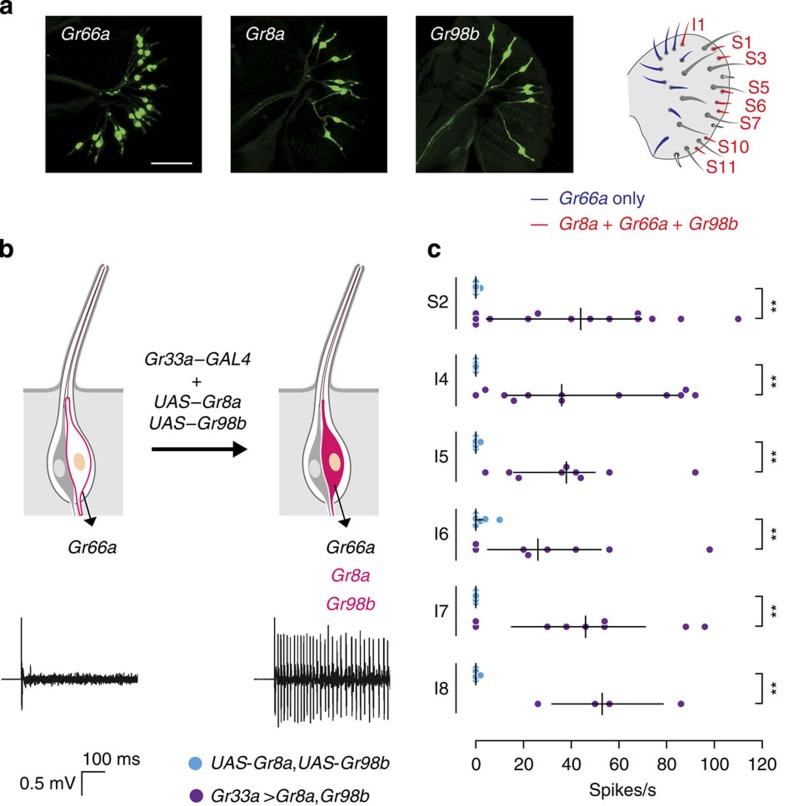
Ectopic *Gr8a* and *Gr98b* expression in bitter-sensing GRNs conferred L-canavanine sensitivity. (**a**) Expression patterns of the *Gr8a*, the *Gr66a* and the *Gr98b* reporters as well as a cartoon indicating the location of *Gr8a*-, *Gr66a*- and *Gr98b*-expressing sensilla in the labellum. The scale bar represents 50 μm. (**b**) Ectopic expression of *Gr8a* and *Gr98b* in bitter-sensing GRNs (I-type sensilla) conferred L-canavanine sensitivity to sensilla that did not normally respond to L-canavanine. A schematic representation of a sensilla depicting the ectopic expression experiment (above) and representative traces (below) evoked by 30 mM  L-canavanine from I4 sensilla of control (*UAS-Gr8a,UAS-Gr98b*) and *UAS–Gr8a*,*UAS–Gr98b*;*Gr33a*–*GAL4* flies. (**c**) Response frequencies evoked by 30 mM L-canavanine after ectopic expression of *Gr8a* and *Gr98b* in the indicated sensilla. *UAS*–*Gr8a*,*UAS*–*Gr98b* flies were the negative controls. *n*=4–14. **P<0.01 (Mann–Whitney *U*-test). Medians and quartiles are shown.

The major taste organ of the fly, the labellum, is decorated with gustatory bristles (sensilla) that fall into three classes based on length and position: long (L), intermediate (I) and short (S)^1^. *Gr66a* is widely expressed in bitter-sensing GRNs of S-type and I-type sensilla^30^,^31^,^32^, whereas *Gr8a* expression is limited to the subset of *Gr66a*-expressing GRNs that respond to L-canavanine^22^. To determine the expression pattern of *Gr98b*, we examined GFP staining in *Gr98b*–*GAL4*;*UAS*–*mCD8::GFP* flies. We detected *Gr98b*–*GAL4* reporter expression in GRNs of I1, S1, S3, S5, S6, S7, S10 and S11 sensilla (Fig. 3a; Supplementary Table 1). Consistent with a role in sensing L-canavanine, this included all of the sensilla that responded to L-canavanine^22^, and the S-type sensilla that contained GRNs that expressed the *Gr8a–GAL4* (refs 22, 30).

To determine whether *Gr8a*, *Gr66a* and *Gr98b* were sufficient to confer L-canavanine sensitivity to GRNs that normally do not respond to L-canavanine, we ectopically expressed *Gr8a* and *Gr98b* in *Gr66a*-expressing GRNs that were insensitive to L-canavanine^22^,^30^,^31^,^32^. Misexpression of *Gr8a* in *Gr66a*-expressing, L-canavanine-insensitive GRNs does not confer L-canavanine sensitivity^22^. We found that introduction of *Gr8a* and *Gr98b* in L-canavanine-insensitive S2- and I-type sensilla under the control of the *Gr33a*–*GAL4* conferred robust L-canavanine responses to these sensilla (Fig. 3b,c). These results indicated that GR8a and GR98b were essential components of the functional L-canavanine receptor.

### Conferring L-canavanine responsiveness to sweet-sensing GRNs

**Figure 4:**
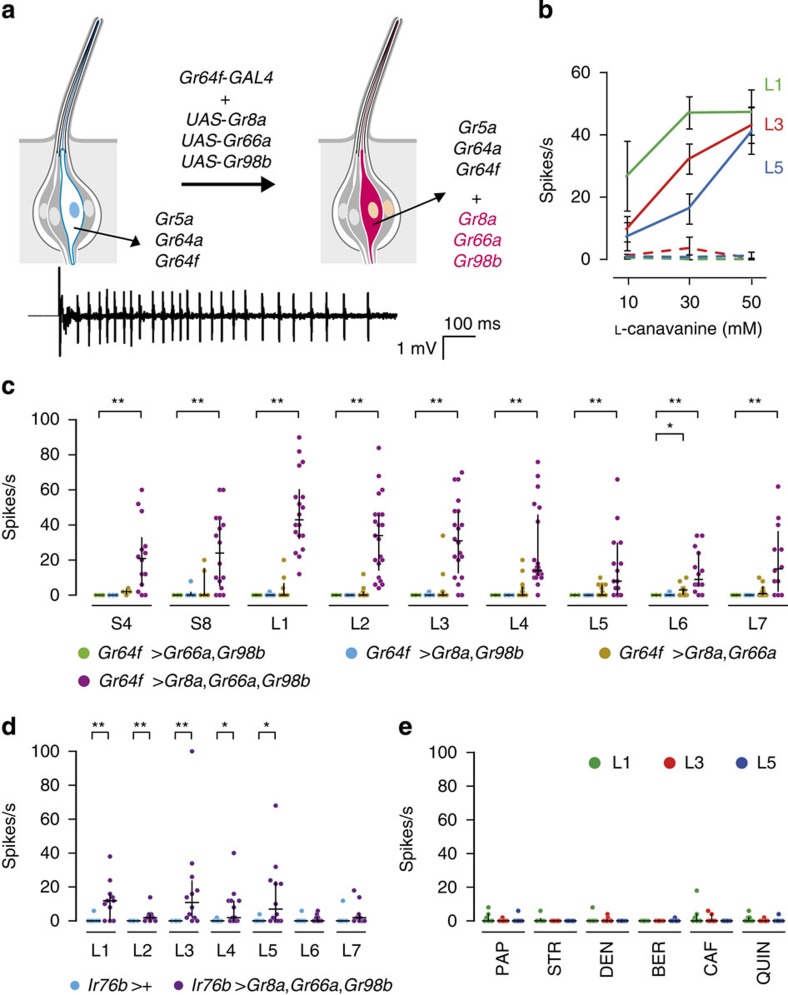
Effects resulting from ectopic expression of *Gr8a*, *Gr66a* and *Gr98b* in sugar and low-salt responsive GRNs. (**a**) Schematic depicting ectopic expression of *Gr8a*, *Gr66a* and *Gr98b* in sweet-sensing GRNs (above) and representative traces (below) evoked by 30 mM L-canavanine from L3 sensilla expressing *Gr8a*, *Gr66a* and *Gr98b* in sweet-sensing GRNs. (**b**) Dose-dependent L-canavanine responses in the indicated sweet-sensing GRNs. Shown are the responses of the indicated sensilla from control flies without a *GAL4* (*UAS*–*Gr8a*,*UAS*–*Gr66a*,*UAS*–*Gr98b*; dashed lines) or flies expressing the three *Gr*s under the control of the *Gr64f*–*GAL4* (solid lines). *n*=6–21. Data are mean±s.e.m. (**c**) Response frequencies evoked by 30 mM L-canavanine after ectopic expression of *Gr8a*, *Gr66a* and *Gr98b* in the indicated sweet-sensing GRNs using the *Gr64f*–*GAL4*. Genotypes: (1) green circles: 2 × (*UAS*–*Gr66a*,*UAS*–*Gr98b*);*Gr64f*–*GAL4*/+, (2) blue circles: 2 × (*UAS*–*Gr8a*,*UAS*–*Gr98b*);*Gr64f*–*GAL4*/+, (3) ochre circles: 2 × (*UAS*–*Gr8a*, *UAS*–*Gr66a*);*Gr64f*–*GAL4*/+ and (4) purple circles: 2 × (*UAS*–*Gr8a*, *UAS*–*Gr66a*,*UAS–Gr98b*);*Gr64f*–*GAL4*/+. *n*=4–21 for each genotype. Shown are the medians and quartiles. *P<0.05, **P<0.01 (Kruskal–Wallis test with Mann–Whitney *U post hoc*-test). (**d**) Response frequencies evoked by 30 mM L-canavanine after ectopic expression of *Gr8a*, *Gr66a* and *Gr98b* in the indicated low salt-sensing GRNs using the *Ir76b*–*GAL4*. Genotypes: (1) blue circles: *Ir76b*–*GAL4*/+, and (2) purple circles: *UAS*–*Gr8a*,*UAS*–*Gr66a*,*UAS*–*Gr98b*/+;*Ir76b*–*GAL4*/+. *n*=8–14. Median and quartile are shown. *P<0.05, **P<0.01 (Mann–Whitney *U*-test). (**e**) Response frequencies from the indicated sensilla in flies ectopically expressing *Gr8a*, *Gr66a* and *Gr98b* in sugar-responsive GRNs. We tested 1 mM of each of the indicated chemicals (BER, berberine; DEN, denatonium; LOB, lobeline; PAP, papaverine; STR, strychnine), except for caffeine (CAF; 5 mM). *n*=6–9. Median and quartile are shown.

Since bitter-sensing GRNs in S2- and I-type sensilla also express other GRs^30^, it is possible that additional avoidance GRs may be involved in L-canavanine detection. Wild-type L-type sensilla contain four GRNs, one of which respond to sugars and not to L-canavanine^22^. To provide stronger evidence that *Gr8a*, *Gr66a* and *Gr98b* were sufficient for L-canavanine sensation, we misexpressed these three *Gr*s in sugar-activated GRNs in L-type sensilla, using the *Gr64f*–*GAL4* (Fig. 4a). We found that this manipulation endowed sweet-sensing GRNs with the ability to respond robustly to L-canavanine (Fig. 4b,c). Ectopic expression of only two of the three GRs was insufficient to confer significant L-canavanine responsiveness (Fig. 4c). However, in 12 out of 90 recordings, co-expression of just two of the *Gr*s (*Gr8a* and *Gr66a*) produced a small L-canavanine-evoked response in L-type sensilla (Fig. 4c). We also ectopically expressed *Gr8a*, *Gr66a* and *Gr98b* in low salt-sensing *Ir76b*–*GAL4*-positive GRNs^33^, and found that this conferred significant L-canavanine responsiveness to these GRNs (Fig. 4d). Ectopic expression of *Gr8a*, *Gr66a* and *Gr98b* did not induce responsiveness to any of several bitter compounds tested (Fig. 4e).

### Eliciting behavioural attraction to L-canavanine

**Figure 5:**
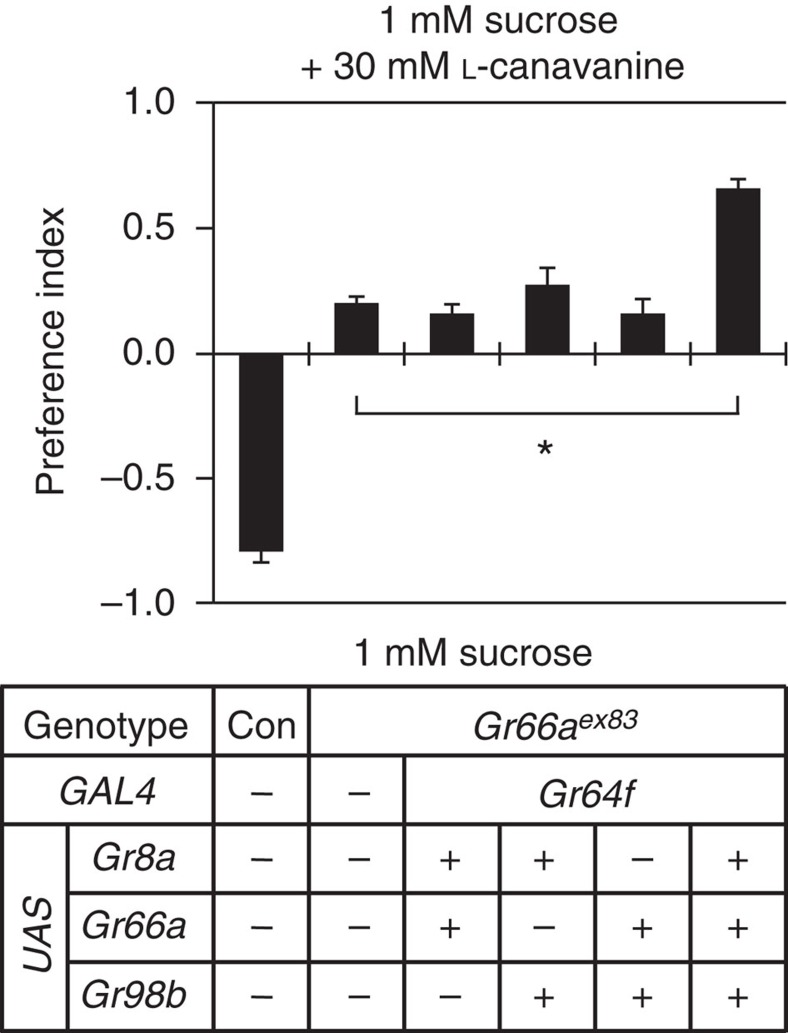
Attraction to L-canavanine induced by ectopic expression of *Gr*s in sugar-sensing GRNs. Two-way choice assays testing for attraction or aversion to L-canavanine in flies misexpressing *Gr8a*, *Gr66a* and *Gr98b* in sweet-sensing GRNs. The *Gr*s were ectopically expressed in a *Gr66a*^*ex83*^ mutant background. *n*=5 for each genotype. Data are mean±s.e.m. *P<0.05 (analysis of variance with *post hoc* Tukey test).

Because expression of *Gr8a*, *Gr66a* and *Gr98b* was sufficient to endow L-canavanine sensitivity to sweet-sensing GRNs, we tested whether ectopic expression of these *Gr*s induced attraction to L-canavanine. Control flies (*w*^*1118*^) strongly preferred 1 mM sucrose over 1 mM sucrose laced with 30 mM L-canavanine (Fig. 5). In contrast, *Gr66a*^*ex83*^ mutants had no preference for either alternative. *Gr66a*^*ex83*^ flies expressing just two *Gr*s (*Gr8a*/*Gr66a*, *Gr8a*/*Gr98b* or *Gr66a*/*Gr98b*) in sweet-sensing GRNs were also nearly indifferent to sucrose alone versus sucrose plus L-canavanine (Fig. 5). However, introduction of all three *Gr*s (*Gr8a*/*Gr66a*/*Gr98b*) induced significant attraction to the L-canavanine-containing food (Fig. 5).

### GR8a/GR66a/GR98b-dependent L-canavanine current in S2 cells

**Figure 6:**
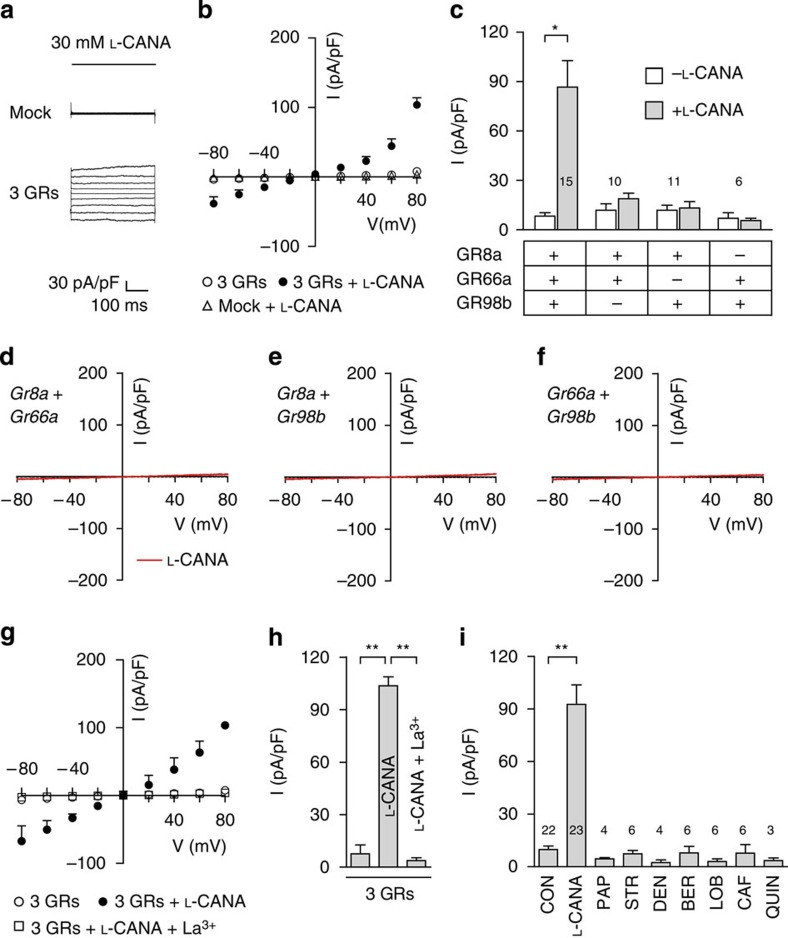
Whole-cell voltage clamp recordings of S2 cells expressing GRs. Cells were stimulated with 30 mM L-canavanine as indicated. (**a**,**b**) Cells were transfected either with pActin5c–*GAL4*, pUAST–*EGFP* only (mock) or pActin5c–*GAL4*, pUAST–*EGFP* plus pUAST–*Gr8a*, pUAST–*Gr66a* and pUAST–*Gr98b* (3 GRs). (**a**) Currents produced in response to voltage steps (−80 mV to +80 mV in 20 mV increments) of 500-ms duration obtained in the presence of L-canavanine. (**b**) I–V relationships using cells expressing GR8a, GR66a and GR98b, and stimulated with L-canavanine. (**c**) Current densities at +80 mV. The cells expressed the indicated GRs and were recorded in the presence or absence of L-canavanine stimulation. The numbers of recordings are indicated. *P<0.05 (paired Student's *t*-test). (**d-f**) Current–voltage traces showing that expression of two GRs in S2 cells did not lead to L-canavanine-induced increases in current densities. (**d**) *Gr8a*- and *Gr66a*-expressing S2 cells (*n*=10). (**e**) *Gr8a*- and *Gr98b*-expressing cells (*n*=11). (**f**) *Gr66a*- and *Gr98b*-expressing cells (*n*=6). (**g**) Effect of La^3+^ on I–V relationship. The cells expressed GR8a, GR66a and GR98b, and were recorded in the presence of L-canavanine. (**h**) Effect of La^3+^ on current densities obtained at +80 mV. The cells expressed the three GRs and were stimulated with L-canavanine. **P<0.01 (analysis of variance (ANOVA) with *post hoc* Tukey test). (**i**) Current densities in cells expressing GR8a, GR66a and GR98b and stimulated with the indicated bitter chemicals: 30 mM L-canavanine, 1 mM papaverine (PAP), 1 mM strychnine (STR), 1 mM denatonium (DEN), 100 μM berberine (BER), 1 mM lobeline (LOB), 5 mM caffeine (CAF), and 1 mM quinine (QUIN). The numbers of recordings are indicated. **P<0.01 (ANOVA with *post hoc* Tukey test). All error bars indicate s.e.m.

To test whether GR8a, GR66a and GR98b could form an L-canavanine-activated cation channel, we co-expressed the three GRs in *Drosophila* S2 cells and performed whole-cell voltage-clamp recordings. We clamped the cells at a holding potential of −60 mV and applied voltage ramps from −80 to +80 mV. Using a normal physiological bath solution, we found that addition of 30 mM L-canavanine produced a slightly outwardly rectifying current with a reversal potential of −0.88±1.89 mV (Fig. 6a,b). However, no current was produced when we expressed any combination of two GRs (GR8a/GR66a, GR8a/GR98b or GR66a/GR98b; Fig. 6c–f). The L-canavanine-induced currents were completely inhibited by La^3+^, a broad spectrum cation channel inhibitor (Fig. 6g,h). Consistent with the *in vivo* data, no other aversive compounds tested induced a conductance in S2 cells expressing GR8a, GR66a and GR98b (Fig. 6i).

## Discussion

Perception of toxic compounds and the aversive behaviors they elicit are innate defense mechanisms shared by many animals, including insects. In *Drosophila*, the detection of most aversive compounds depends on members of the GR family^19^,^20^,^21^,^22^,^23^,^30^. The subunit composition of GR receptors is complicated. Consequently, a major challenge in the field has been to define the repertoire of subunits that are sufficient to render a functional bitter receptor *in vivo* and *in vitro*.

We and others have made multiple attempts to elucidate the composition of a functional receptor complex that senses a repulsive compound^21^,^23^,^30^,^34^. However, none of these undertakings have been entirely successful. For example, *Gr33a*, *Gr66a* and *Gr93a* are essential for detecting caffeine, but misexpression of these GRs in sweet-sensing GRNs is insufficient to confer sensitivity to caffeine^23^. Similarly, we have defined several GRs that contribute to DEET sensation by GRNs^19^. However, ectopic expression of these GRs in non-DEET-responsive GRNs is not adequate to elicit DEET sensitivity^19^. Ectopic expression of *Gr59c* in *Gr21a*/*Gr63a* CO_2_-sensing neurons or in a subset of bitter-sensing GRNs that do not normally respond to berberine, denatonium and lobeline confers sensitivity to these tastants^30^,^34^. Nevertheless, GR59c has not been functionally expressed *in vitro*, and so it remains unclear whether it forms a homomeric or heteromeric receptor. Consistent with the latter possibility, misexpression of *Gr59c* in sweet-sensing GRNs is ineffective, indicating that one or more additional GRs are required for detection of these avoidance compounds^30^.

We found that *Gr8a*, *Gr66a* and *Gr98b* were sufficient to generate a functional L-canavanine-sensing GR complex *in vivo* in every type of GRN tested that was formerly unresponsive to L-canavanine. These included low salt-sensing and sweet-sensing GRNs. Moreover, activation of sweet GRNs with L-canavanine, after misexpressing the three *Gr*s, converted the natural aversion to L-canavanine to attraction^35^,^36^,^37^. Because low-salt and sweet GRNs do not normally express any aversive GR, our findings indicate strongly that there are no additional GRs that comprise the L-canavanine receptor.

The combination of GR8a and GR66a resulted in a low level of L-canavanine sensitivity in a few GRNs that do not normally respond to L-canavanine. Thus, GR8a and GR66a may produce a low-affinity L-canavanine receptor, while GR98b is required for a high-affinity receptor. The GR heteromultimers required for sensing other aversive tastants may be even more complex than the three GR subunits that are required and sufficient for responding to L-canavanine. Because ectopic expression of the three GRs known to be required for responding to caffeine or the three GRs essential for tasting DEET are insufficient for conferring responses to these compounds^19^,^21^, these assemblies appear to be comprised of at least four GRs subunits.

The minimum number of GR subunits critical for sensing attractive tastants remains unresolved. Ectopic expression of single GRs in the CO_2_-sensing olfactory neurons endows these cells with glycerol and sugar sensitivities, raising the possibility that homomeric GRs elicit these responses^34^,^38^. However, CO_2_ neurons express GR21a, GR63a^39^ and possibly other GRs that could potentially form heteromultimeric GR complexes. Mutational analyses and unsuccessful ectopic expression studies indicate that most sugar receptors consist of a complexity of subunits^40^,^41^,^42^. The notable exceptions are insect fructose receptors, which function as ligand-gated cation channels in HEK293 cells^18^.

We demonstrated that introduction of GR8a, GR66a and GR98b *in vitro* in *Drosophila* S2 cells conferred an L-canavanine-induced cation conductance. Thus, we conclude that these three GRs comprise a heteromultimeric L-canavanine-activated channel, which is required and sufficient for detecting this aversive compound in GRNs. This three subunit channel is more complex than the channels formed by the distantly related ORs, which are comprised of just two subunits^15^,^16^. One of the two OR subunits is a broadly required olfactory co-receptor, which is essential for trafficking of the OR complex^43^. Similarly, since GR66a is essential for sensing a broad array of aversive tastants^19^, it may also represent a co-receptor that participates in trafficking of other GRs, such as GR8a and GR98b, which provide tastant specificity.

Finally, activation of the L-canavanine cation conductance in GRNs inhibits feeding in *Drosophila*. Other Dipterans such as mosquitoes feed on human hosts and in doing so, spread prevalent diseases, including malaria and Dengue fever. The discovery that a *Drosophila* GR receptor complex that responds to a repulsive compound is a cation channel, offers the possibility of finding effective compounds to suppress feeding by insect disease vectors, by performing high-throughput screens for activators of similar complexes that function in mosquitoes.

## Methods

### Fly stocks

All fly stocks were maintained on conventional cornmeal–agar–molasses medium, with 12-h light/12-h dark cycles at 25 °C and 60% humidity. We obtained the following fly stocks from the Bloomington Stock Center: (1) *70FLP*,*70I-SceI*/*CyO*, (2) *UAS*–*Dcr*2 and (3) *UAS*–*mCD8*::*GFP*. The *UAS*–*Gr8a*, *UAS*–*Gr66a*, *Gr33a*–*GAL4* and *Gr8a*–*GAL4* flies were described previously^20^,^21^,^22^,^30^. The *Gr66a*–*GAL4* flies were a gift from H. Amrein^32^. *Gr98b*–*GAL4* and *Gr64f*–*GAL4* were provided by J. Carlson^30^,^44^. We obtained the fly stocks for the RNAi screen from the Vienna Drosophila RNAi Center and the Bloomington Stock Center. The stock numbers are as follows: *Gr2a* (v102185), *Gr5a* (v13730), *Gr8a* (v31104), *Gr9a* (v15446), *Gr10a* (v39237), *Gr10b* (v31151), *Gr21a* (v104122), *Gr22a* (v106736), *Gr22b* (v107792), *Gr22c* (v7249), *Gr22e* (v9389), *Gr22f* (v102860), *Gr23a* (v40852), *Gr28a* (v100938), *Gr28b* (v101727), *Gr32a* (v47956), *Gr33a* (v42802), *Gr36a* (v48018), *Gr36b* (v8062), *Gr36c* (v3872), *Gr39a* (v8685), *Gr39b* (v33215), *Gr43a* (v39518), *Gr47b* (v4594), *Gr57a* (v45879), *Gr58a* (v1703), *Gr58b* (v9565), *Gr58c* (v29137), *Gr59a* (v31107), *Gr59b* (v101219), *Gr59c* (v3530), *Gr59d* (v2766), *Gr59e* (v31110), *Gr59f* (v18989), *Gr61a* (v106007), *Gr63a* (v108203), *Gr64a* (v103342), *Gr64b* (v42517), *Gr64c* (BL36734), *Gr64d* (v29422), *Gr64e* (v109176), *Gr64f* (v105084), *Gr66a* (v14820), *Gr68a* (v13380), *Gr77a* (BL38236), *Gr85a* (v47992), *Gr89a* (v8253), *Gr92a* (v44408), *Gr93a* (v13569), *Gr93b* (v12160), *Gr93c* (v109794), *Gr93d* (v6813), *Gr94a* (v9537), *Gr97a* (v4395), *Gr98a* (v1300), *Gr98b* (v1302 and v101040), *Gr98c* (BL36735), *Gr98d* (v4398), *IR7a* (v108171), *IR47a* (v11812), *IR56a* (v5010), *IR56b* (v4704), *IR56d* (v6112), *IR94e* (v33066), *IR20a* (v8658), *IR94a* (v7566), *IR94c* (v6817), *IR94h* (v1563), *IR60b* (v12089), *IR67c* (v37261) and *IR94f* (v109702).

### Genetics

We generated the *Gr98b* mutant (*Gr98b*^*1*^) by ends-out homologous recombination. To obtain the DNA construct for the homologous recombination, we used a genomic DNA template from isogenic *w*^*1118*^ flies and PCR to amplify 3 kb arms located 5′ and 3′ to the targeted *Gr98b* locus. The primers used for the 5′ arm were 5′-GGTGGCTTAGGTGCTGCCATTAC-3′ and 5′-TTGGGTGAGTTCTGAAAACTAAC-3′. The primers for the 3′ arm were 5′-TCTGAAACGCAATCAATTGCTA-3′ and 5′-GTAGCCCAATATCACAATTC-3′. We subcloned the two arms into the pw35 vector^45^, the transgenic flies were generated by germline transformation (BestGene, Inc., Chino Hills, CA), and the transgene was mobilized to generate the homologous recombinants as described^45^. We confirmed the *Gr98b*^*1*^ allele via genomic PCR, in conjunction with the following primers: 5′-TCTCCTGGCCAGAGCCTTTCCATA-3′ and 5′-TGCTGCATTATCATGACGAACTCGG-3′.

To generate the *UAS*–*Gr98b* transgenic flies, we amplified a *Gr98b* cDNA from a *w*^*1118*^-derived labellar cDNA library using the Hi-fidelity PCR kit (Roche), and cloned the cDNA into the pUAST vector. We verified the cDNA clone by DNA sequencing and the transgenic flies were generated by BestGene, Inc. We outcrossed the *Gr98b*^*1*^ and the *UAS*–*Gr98b* flies to *w*^*1118*^ flies for five generations.

### Imaging

We performed immunostaining of whole mount fly labella^21^ using rabbit anti-green fluorescent protein (GFP) (1:1,000, Molecular Probes) primary antibodies and goat anti-rabbit Alexa488 (1:400, Molecular Probes) secondary antibodies. The labella were dissected from heads, fixed for 20 min using 4% paraformaldehyde diluted in PBS-T (1 × PBS for 20 min and 0.2% TritonX-100) and washed three times with PBS-T. The labella were bisected with a razor blade, incubated for 30 min in blocking solution (5% heat-inactivated goat serum in PBS-T) and incubated overnight at 4 °C with the primary antibodies diluted in the blocking solution. The tissues were washed three times with PBS-T and incubated with the secondary antibodies diluted in blocking solution for 1 h at room temperature. Following three washes with PBS-T, the samples were mounted with Vectashield (Vector Laboratories, Burlingame, CA) and visualized with a Zeiss LSM700 confocal microscope (Jena, Germany).

### Chemicals

Sucrose, denatonium, quinine, papaverine, caffeine, strychnine, L-canavanine, N,N-diethyl-m-toluamide (DEET), sulforhodamine B, KCl and tricholine citrate were purchased from Sigma-Aldrich (Saint Louis, MO). Berberine sulfate trihydrate and Brilliant Blue FCF were obtained from Wako Pure Chemical Industries, Ltd (Osaka, Japan).

### Two-way choice behavioural assay

The binary food choice assays were performed in a blinded fashion as described previously^11^,^20^. Briefly, for each assay we starved ∼50 (3–6 days old) flies for 18 h and placed them in 72-well microtiter dishes. Each alternating well was filled with 1% agarose combined with one of the two types of test mixtures. The aversion to bitter chemicals was assayed by comparing the preferences for 1 mM sucrose to 5 mM sucrose plus the indicated concentrations of aversive compounds. To measure the effect of activation of sugar GRNs by L-canavanine, we tested the preference for 1 mM sucrose versus 1 mM sucrose plus 30 mM L-canavanine.

To monitor food intake, we added blue dye (Brilliant Blue FCF, 0.125 mg ml^−1^) to one test mixture, and red dye (sulforhodamine B, 0.2 mg ml^−1^) to the other. After allowing the flies to feed for 90 min at room temperature in the dark, the animals were frozen at −20 °C. The numbers of blue (N_B_), red (N_R_) or purple (N_MIX_) flies were counted under a dissection microscope and the preference index (PI) values were calculated according to the following equation: (N_B_−N_R_)/(N_R_+N_B_+N_MIX_) or (N_R_−N_B_)/(N_R_+N_B_+N_MIX_). PIs of 1.0 and −1.0 indicate complete preference for one or the other food. A PI of 0 indicates no preference.

### Tip recordings

Tip recordings^46^ were performed as we described previously^20^. Following eclosion, we maintained flies on fresh food for 1 day. We immobilized the animals by inserting a glass capillary reference electrode filled with Ringer's solution into the abdomen, and extending it to the head. We stimulated labellar sensilla with recording electrodes (10–20 μm tip diameter) containing tastants dissolved in 1 mM KCl or 30 mM tricholine citrate. The recording electrode was connected to a preamplifier (TastePROBE, Syntech, Hilversum, The Netherlands) and taste responses were collected and amplified (10 ×) using a signal interface (Syntech) in conjunction with a 100–3,000 Hz band-pass filter. The inputs were also linked to a loudspeaker to facilitate audio monitoring. We recorded action potentials at a 12-kHz sampling rate, sorted the spikes based on amplitude, and performed quantification using Autospike 3.1 software package (Syntech).

### Cell culture and transfection

We grew S2 cells in Schneider's Insect media (Welgene, Gyeongsan-si, Republic of Korea) supplemented with 10% fetal bovine serum (Invitrogen, Carlsbad, CA), 50 units per ml penicillin-streptomycin (Invitrogen) in T-25 flasks (Thermo, Waltham, MA) at 25 °C. To perform the patch clamp experiments, we transfected cells 24 h after plating with pActin5c–*GAL4*, pUAST–*EGFP* (enhanced green fluorescent protein) and the two or three of the following *Gr* plasmids using X-tremeGENE HP DNA transfection reagent (Roche): pUAST–*Gr8a*, pUAST–*Gr66a* and pUAST–*Gr98b*. The transfection mixture consisted of 4 μl of transfection reagent and 1.3 μg of total DNA. After incubating the cells with the transfection cocktail in serum-free media for 12 h, we switched to serum-containing media, and continued to incubate the cells for 24 h to allow for expression of the GRs and EGFP.

### Patch clamp experiments in S2 cells

We transferred *Gr*- and *EGFP*-expressing S2 cells on coverslips to a chamber positioned on the stage of an inverted microscope (IX71, Olympus). Whole cell currents were measured using an Axon 200B amplifier at a holding potential of −60 mV. The bath solution contained normal Ringer's solution (in mM): 140 NaCl, 5 KCl, 5 HEPES, 2 pyruvic acid sodium salt, 1.25 KH_2_PO_4_, 2 CaCl_2_, 2 MgCl_2_ and 10 D-glucose (pH 7.4). The pipette solution contained (in mM): 140 KCl, 5 EGTA-2 K, 10 HEPES and 10 D-glucose (pH 7.2). We pulled electrodes from borosilicate glass that had resistances of 2–4 MΩ after fire polishing. The seal resistances were between 3 and 10 GΩ. After establishing a whole-cell configuration, we recorded currents in the presence of L-canavanine, by applying hyperpolarizing and depolarizing voltage pulses using a holding potential of −60 mV and voltage ramps from +80 and −80 mV in steps of 20 mV. We performed all recordings at room temperature using an Axopatch-200B amplifier (Axon Instruments, Foster City, CA). We digitized the currents with a Digidata 1440 A converter (Axon Instruments) filtered at 5 kHz. Command potential and data acquisition were controlled with pClamp 10.2 software (Axon instruments). Whole-cell recording data analyses were performed using Clampfit 10.2. The current densities were normalized to the cell capacitance.

### Statistical analyses

We performed statistical analyses using the SPSS 21.0 (IBM Corporation, Armonk, NY). All data, except for ectopic expression of GRs, were analysed using unpaired Student's *t*-tests for comparing two sets of data or one-way analysis of variance with Tukey *post hoc* tests for comparing multiple sets of data, as these data passed the Kolmogorov–Smirnov test. The data represent the means±s.e.m.

Electrophysiological data for misexpression of *Gr*s were analysed using non-parametric tests (Figs 2c and 3c,d). We employed the Mann–Whitney *U*-test for comparing two sets of data. We performed a Kruskal-Wallis test with Mann–Whitney *U post hoc*-test to determine whether the medians of two genotypes were significantly different. The data presented are the medians and quartiles.

## Additional information

**How to cite this article:** Shim, J. *et al*. The full repertoire of Drosophila gustatory receptors for detecting an aversive compound. *Nat. Commun.* 6:8867 doi: 10.1038/ncomms9867 (2015).

## Supplementary Material

Supplementary Information Supplementary Table 1 and Supplementary References
