# Supplementary material for: The full repertoire of Drosophila gustatory receptors for detecting an aversive compound
Source: Nat Commun. 2015 Nov 16;6:8867. doi: 10.1038/ncomms9867 (PMC4660205; doi:10.1038/ncomms9867)
Supplement: Supplementary Information — Supplementary Table 1 and Supplementary References [file ncomms9867-s1.pdf]

Supplementary Table 1

Sensillum nomenclature	Tanimura[1]	I1	S1	S3		S5	S6	S7	S10	S11
	Carlson[2]	S0	S1	S2	S3	S5	S6	S7	S9	S10
Gr98b-GAL4 expression	This study	8/9	9/9	6/9		5/9	9/9	5/9	8/9	9/9
	Weiss *et al.*[2]	0.75	0.63	0.61	0.19	0.17	0.88	0.24	0.28	0.92

Supplementary Table 1. Expression of the *Gr98b-GAL4* reporter in sensilla in the labellum

Two independent sensillum nomenclature systems have been employed[1,2]. In this study, we used the system described by the Tanimura group[1]. We examined GFP signals in the labellum of *Gr98b-GAL4*;*UAS-mCD8::GFP* flies after immunostaining with GFP antibodies. Listed are the proportions of GFP expressing GRNs (This study). Shown below are previously reported *Gr98b-GAL4* reporter expression described by Weiss *et al.*[2]. According to their system, 1.0, 0.5 and 0 corresponded to positive expression, weak expression, and no expression, respectively[2].

Supplementary References

1 Hiroi, M., Marion-Poll, F. & Tanimura, T. Differentiated response to sugars among labellar chemosensilla in *Drosophila*. *Zoolog. Sci.* **19**, 1009-1018 (2002).

2 Weiss, L. A., Dahanukar, A., Kwon, J. Y., Banerjee, D. & Carlson, J. R. The molecular and cellular basis of bitter taste in *Drosophila*. *Neuron* **69**, 258-272 (2011).
